# Extracting prime protein targets as possible drug candidates: machine learning evaluation

**DOI:** 10.1007/s11517-023-02893-0

**Published:** 2023-08-23

**Authors:** Subhagata Chattopadhyay, Nhat Phuong Do, Darren R. Flower, Amit K. Chattopadhyay

**Affiliations:** 1 Dept. of Computer Science and Engineering, GITAM School of Technology, Gandhi Institute of Technology And Management (GITAM) deemed to be University, Bengaluru, Karnataka 561203 India; 2 Department of Applied Mathematics and Data Science, College of Engineering and Physical Sciences, Aston University, Birmingham, B4 7ET UK; 3 School of Life and Health Sciences, Aston University, Birmingham, B4 7ET UK

**Keywords:** Molecular docking, Protein–ligand interaction, Drug design, Ligands, Protein targets, Data mining, Machine learning (ML), K-means clustering, Gaussian mixture model, DBSCAN, DUD-E repository, Forward modeling, Reverse modeling

## Abstract

Extracting “high ranking” or “prime protein targets” (PPTs) as potent MRSA drug candidates from a given set of ligands is a key challenge in efficient molecular docking. This study combines protein-versus-ligand matching molecular docking (MD) data extracted from 10 independent molecular docking (MD) evaluations — ADFR, DOCK, Gemdock, Ledock, Plants, Psovina, Quickvina2, smina, vina, and vinaxb to identify top MRSA drug candidates. Twenty-nine active protein targets (APT) from the enhanced DUD-E repository (http://DUD-E.decoys.org) are matched against 1040 ligands using “forward modeling” machine learning for initial “data mining and modeling” (DDM) to extract PPTs and the corresponding high affinity ligands (HALs). K-means clustering (KMC) is then performed on 400 ligands matched against 29 PTs, with each cluster accommodating HALs, and the corresponding PPTs. Performance of KMC is then validated against randomly chosen head, tail, and middle active ligands (ALs). KMC outcomes have been validated against two other clustering methods, namely, Gaussian mixture model (GMM) and density based spatial clustering of applications with noise (DBSCAN). While GMM shows similar results as with KMC, DBSCAN has failed to yield more than one cluster and handle the noise (outliers), thus affirming the choice of KMC or GMM. Databases obtained from ADFR to mine PPTs are then ranked according to the number of the corresponding HAL-PPT combinations (HPC) inside the derived clusters, an approach called “reverse modeling” (RM). From the set of 29 PTs studied, RM predicts high fidelity of 5 PPTs (17%) that bind with 76 out of 400, i.e., 19% ligands leading to a prediction of next-generation MRSA drug candidates: *PPT2* (average HPC is 41.1%) is the top choice, followed by *PPT14* (average HPC 25.46%), and then *PPT15* (average HPC 23.12%). This algorithm can be generically implemented irrespective of pathogenic forms and is particularly effective for sparse data.

**Graphical Abstract:**

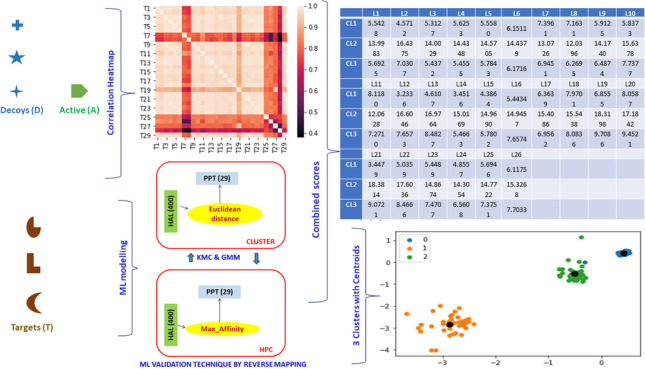

## Introduction

Drug design is a key aspect of healthcare that relies on accurate identification of biologically active substances from protein targets (PT) [1]. Ligands (Ls) comprise such biologically active substances that control PTs, which are the functional biomolecules used in the processes of cellular transduction, transformation, and conjugation [2], and hence pharmacokinetic response of the active ligands [3]. PTs can be composed of ion channels, receptors, enzymes, or porter molecules with which drugs-like-ligands bind [2]. Detecting successful L-PT combinations, or more specifically high affinity ligands (HALs) with prime protein targets (PPTs), is still a challenge as new diseases are continuously emerging that require fast responding, high efficacious new drugs with lower adverse effects that are budget conducive as well. The present extensively interdisciplinary study combines tools drawn from molecular biology, probabilistic mathematics, and computer science to automate the detection of HAL and PPTs from the best ligand–protein combinations to identify next-generation MRSA drug candidates.

MRSA is a bacterial infection that is resistant to several antibiotics, making it difficult to treat. The development of AI-powered drugs has offered new hope in the fight against MRSA. Current state of MRSA drugs using artificial intelligence (AI): AI-powered drugs have shown great promise in the fight against MRSA. AI have been used to identify new compounds that can attack MRSA bacteria, and these compounds have been tested in clinical trials. One such compound is called LFF571, which has shown promising results in treating MRSA infections. AI-powered drugs have the potential to revolutionize the way we treat MRSA and other antibiotic-resistant infections. By using AI to identify new compounds, scientists can develop drugs that are more effective and have fewer side effects. AI can also help to identify new drug targets, which can lead to the development of more targeted therapies.

The present study targets three key areas of MRSA drug designing: (i) computational extraction or detection of HAL for PTs, (ii) computational extraction of PPT for HALs, and (iii) probabilistic prediction of interactions of new PTs and Ls [4]. This work primarily focuses on identifying the top PPTs for the corresponding HALs. The novelty lies in stockpiling molecular docking data from 10 different architecture (ADFR; DOCK; Gemdock; Ledock; Plants; Psovina; Quickvina2; smina; vina; and vinaxb) that independently analyze different biochemical pathways, and then combining them using machine learning, first to dimensionally reduce the key elements and then to regress towards probabilistic predictive models.

The study combines information from several machine learning (ML) algorithms to identify correct L, PT candidates, and combinations of two (popularly called as *structure–activity-relationship* or *SAR* or quantitative structure–activity-relationship or *QSAR*) at the outset of a drug design [5]. In SAR, from the structural features of the compound, its biological activities are predicted. SAR is also able to predict the combinatorial strength of the new composite compound benchmarked on a set of pre-trained compounds whose activities are already tested. However, its limitation is noted in L-PT interactions. SAR is unable to predict PT if the Ls are unknown [4]. Therefore, efforts have been made to solve this issue with L-PT 3-D modeling [6]. This approach is not free of its own limitation either. Firstly, L-PT-3D requires knowledge of the full 3-D protein structure, which is not always feasible. Secondly, it relies on an extensive chemical library, and relatively heavy computation [4]. To address these issues, researchers used a sequence of supervised learning algorithms, known as “proteochemometrics,” that outline classifiers that can predict Ls and PTs individually and jointly in a combined formation [7]. These classifiers are support vector machines (SVMs), regressions, artificial neural networks (ANN), fuzzy classifications, and so forth as promising predictors for successful identification of drug targets [8, 9]. K-means clustering (KMC) has also been tried in several studies to discover candidate proteins and its corresponding high affinity agents, particularly in functionality mapping of candidate proteins [10]. Given that we have a phenomenological idea as to the number of clusters and the cluster centers, K-means is an ideal choice for us initially, and then, we validate the performance of KMC with two more clustering techniques, Gaussian mixture model (GMM) and density-based spatial clustering of applications with noise (DBSCAN).

This study automates the extraction of PPTs for a given sample with HALs, initially using data mining and data modeling (DDM), called “forward modeling” (Approach I), and then using a KMC-based “reverse modeling” approach (Approach II) to automate and validate the observations from forward modeling. We later validate the performance of KMC with GMM and DBSCAN, as mentioned. This allows for a statistical estimation within the constraints of sparse data, an approach that can substantially reduce the time needed to find PPTs, thus substituting rigorous laboratory experiments, and hence in optimizing the resources involved with wet-lab experiments.

The next sections illustrate the methodology adopted, demonstration and explanation of the results, and generic implementations of the methodology in drug development studies.

## Methodology

In this section, we first explain the composition of the DUD-E data (http://dude.docking.org/), and how approaches I and II detailed below can be used to analyze these data.

- Approach I: DMM called “forward modeling.” The aim is to mine HALs and its corresponding PTs.
- Approach II: K-means clustering as machine learning (ML) technique to automate the prediction of PPTs and validate the HAL-PT combinatorial models thus obtained from the experiments of Approach I (called “reverse modeling”). The performance of KMC is further validated by GMM and DBSCAN clustering methods.

### DUD-E data

Tier 1 involves docking data from the enhanced DUD-E repository (http://dude.docking.org/) using 10 popular and easily accessible (open access) docking programs — ADFR, DOCK6, Gemdock, Ledock, PLANTS, PSOV-ina, QuickVina2, Smina, Autodock Vina, and VinaXB. The choice is governed by reported individual success rates, e.g., DOCK6 at 73.3% [11], Autodock Vina at 80% [12], Gemdock at 79% [13], ADFR at 74% [14], Ledock at 75% [15], PLANTS 72% [16], PSOVina 63% [17], QuickVina2 63% [18], Smina more than 90% [19], and VinaXB 46% [20]. Tier 2 combines data from all 10 scores using statistical (linear and nonlinear) models belonging to four universality classes (detailed later). Tier 3 is about normalizing VS enhancement data from Tier 2 through a novel calibration of the individual best score (Smina in our case) against the respective probability density functions (PDF); existence of Tier 2 PDF points beyond the best individual score defining the improved docking performance from the algorithm in Tier 2. PDF data being non-dimensional, normalization is guaranteed and that too without any information loss. A recent statistical study from our group [21], structured on the ubiquitous consensus scoring (CS) approach, has analyzed the same docking data [11–20] to outline a substantially less computationally demanding structure to identify top PPT candidates, starting from a statistical mechanics-based universality class approach. Apart from establishing improved ligand–protein docking fidelity through this approach, the study will also serve as a validity benchmark of the ML-based present approach. As shown later, the ML approach compares favorably with its CS counterpart.

Each DUD-E database (DB) consists of 1040 ligands (L) × 29 protein target (PT). Out of 1040, 1000 are decoy ligands (DL), i.e., inactive, and 40 are active ligands (AL). “Decoys” are therefore discarded, and “actives” are considered for the study. Each L has its “affinity” towards a corresponding PT. Ligand–protein binding (LPB) or docking occurs only when the change in the Gibbs free energy of the system is “negative” when the system reaches its thermodynamic equilibrium at a constant pressure and temperature. Therefore, “negative” affinities denote successful LPB/docking. As the extent of LPB/docking is determined by the magnitude of the said negative energy, it can be safely suggested that the magnitude of the negative affinity determines the stability of any ligand protein complex (LPC).

Each ligand in a DB is considered “unique,” that is, the same ligand (similar affinities to corresponding PTs) never recurs in any other DB under consideration.

A representative data matrix is shown in Table 1 below. It shows the affinity strengths (cell values) of the first 4 Ls corresponding to the 29 PTs in ADFR. Note that affinities are “negative” in numbers, indicating attractive potential. Similar AL-PT combinations for the remaining 9 DBs are extracted.

**Table 1:**
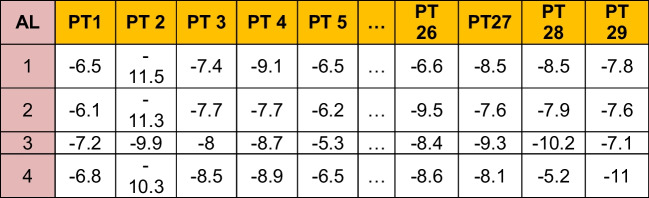
Sample of a DB

### Approach I: data mining and data modeling (DDM) — ‘forward Modeling’

The key objective here is to extract HALs from the unlabeled cluster data and identify the probabilistically matching PPT for successful molecular docking with respect to successful drug design.

#### Data mining steps (carried out for each DB)

A) Identification of HALs and extracting the corresponding PTs based on *affinity maxima*. Essentially, it is the measure/magnitude of HALs.
B) Grouping proximal HAL candidates based on Euclidean distance (ED) separation of similar or close to *maximum affinity* within each DB.
C) Finding ligands (Ls) with highest overall affinities by calculating the *maximum of the mean affinity* across all PTs and its *spread (maximum of the standard deviation* across PTs. It can be stated that such an L or a group of Ls show high affinity towards all PTs and thereby accommodate maximum PTs during DOCKING.
D) Finding *most receptive PTs* that can bind with the maximum number of HALs, *computing column-wise high affinities*.
E) Tabulating percentages of HALs amongst total ligands.

Details of the observations are mentioned below.

#### Summary steps of DDM (DB-wise)

Table 2 shows the HALs, their respective high affinities, corresponding PPTs, the (global) maximum of the mean affinities of HALs, affinity standard deviation, and percentages of HAL contributions in tabular format for the given DB. Maximum of the mean affinities identifies the L with the overall highest binding capacity calibrated against the dispersion (i.e., standard deviation) of the affinities around mean. The highest ranked HALs are marked in blue.

**Table 2:**
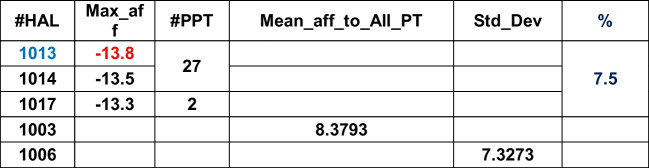
ADFR

HAL1013, 1014, and 1017 (3/40, i.e., 7.5%) have affinities close to each other and therefore considered as effective Ls. While HAL1013 and HAL1014 show closer affinity towards PT27, the HAL 1017 affinity maps against PT2. PT27 and PT2 are thus called prime PTs (PPTs). HAL1003 and HAL1006 show most overall affinities to bind with all PTs. The HPC, mean, and standard deviations for the remaining DB are shown below in Tables 3, 4, 5, 6, 7, 8, 9, 10 and 11.

**Table 3:**
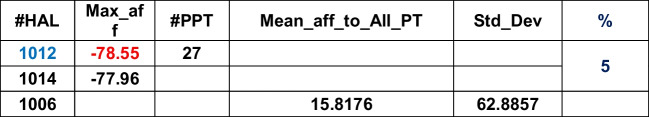
DOCK

**Table 4:**
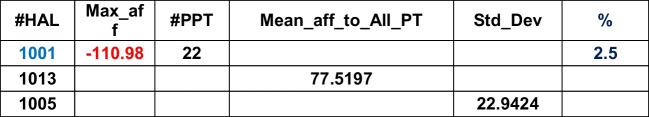
Gemdock

**Table 5:**
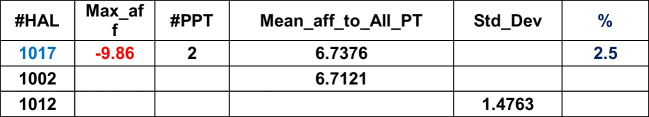
Ledock

**Table 6:**
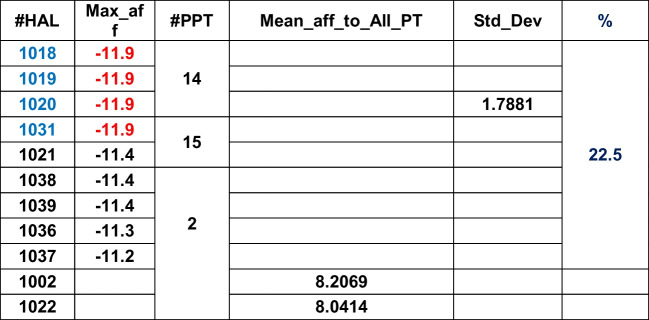
Plants

**Table 7:**
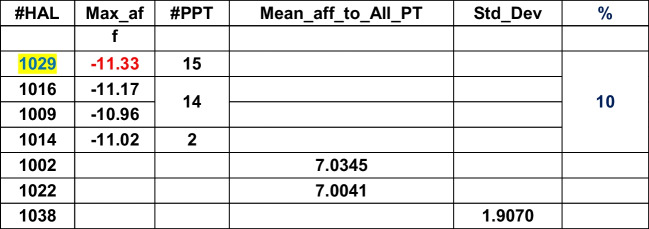
Psovina

**Table 8:**
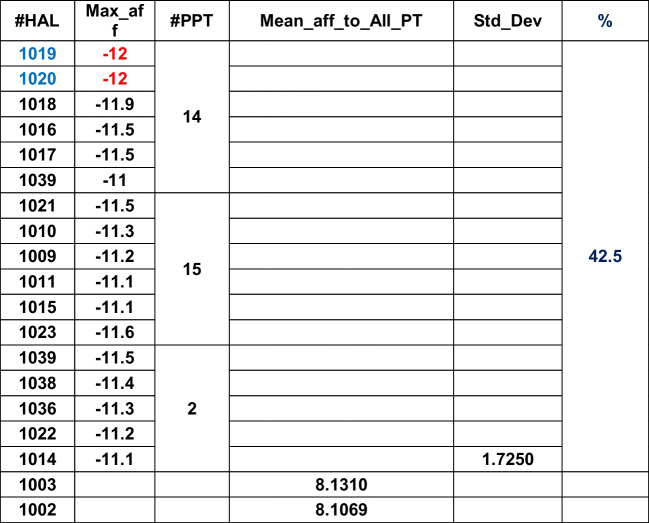
Quickvina2

**Table 9:**
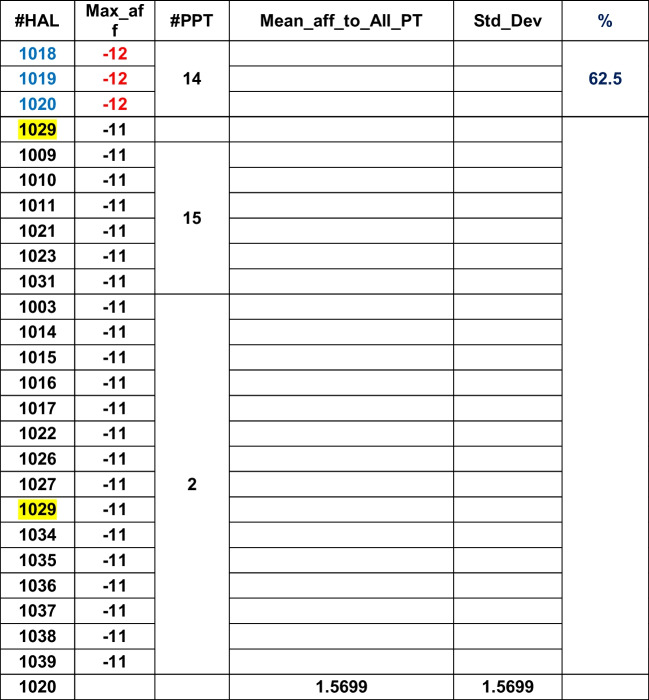
Smina

**Table 10:**
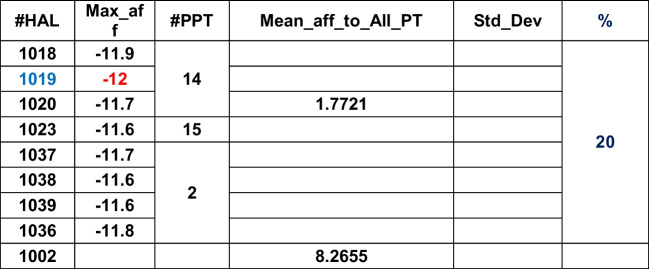
Vina

**Table 11:**
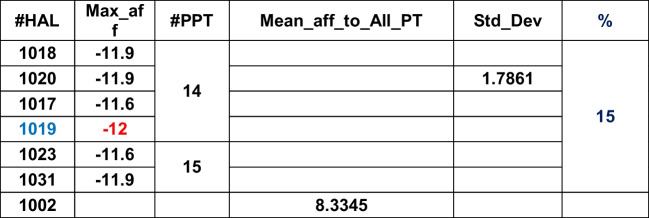
Vinaxb

In the *plants* DB, HALs show redundancies in the magnitude of affinities. In the final ranking, such redundancies are removed.

#### Summary of DDM

A) HALs (blue font) with “high” affinities to the corresponding PTs are obtained DB-wise and shown in Tables 2, 3, 4, 5, 6, 7, 8, 9, 10 and 11. Highest affinity can be seen in Gemdock (HAL1001, affinity magnitude − 110.98, showing affinity to bind with PPT22). Due to its high magnitude, it is an outlier. Discarding it will amount to key information loss. Hence, we use a machine learning (ML) technique (KMC) to accommodate such extremal values on scalar data.
B) DB that is able to provide maximum information on HPC is Smina (62.50%), followed by Quickvina2 (42.5%). Plants (22%) is the third rank holder.
C) AL with the highest overall affinity (mean affinity across its values) is 1002.
D) Ligand with the highest overall affinity towards all 29 target proteins is 1013 (mean 77.51).
E) Ligand with the highest accommodation across all 29 target proteins is 1006 (standard deviation 62.88).
F) Ligand 1029 is the most versatile as it can bind with target proteins 2 (affinity − 11), 14 (affinity − 11), and 15 (affinity − 11.33).
G) Total number of ligands with high affinity is 76 out of 400, i.e., 19%. After redundancy check (i.e., eliminating ligands with similar affinities), final number of ligands with high affinity is 22 out of 400, i.e., 5%. After redundancy check, the relative percentage of HALs against the PPTs show as follows — with PPT2 (55%), PPT14 (19%), PPT15 (4%), PPT27 (18%), and PPT22 (4%). Overall, out of 29, only 5 PTs (17%) show high receptiveness towards these ligands. This information is crucial to create the DUD-E data mining and data modeling (DDM). After redundancy check, DB that have contributed in extracting maximum information are ADFR (rank1), DOCK (rank2), Gemdock (rank3), Ledock (rank4), Plants (rank5), Psovina (rank 6), Quickvina2 (rank 7), and Vina (rank 8).
H) After *max–min normalization*, the affinities are shown under “Norm_Affinity” column in Table 12 below.

**Table 12:**
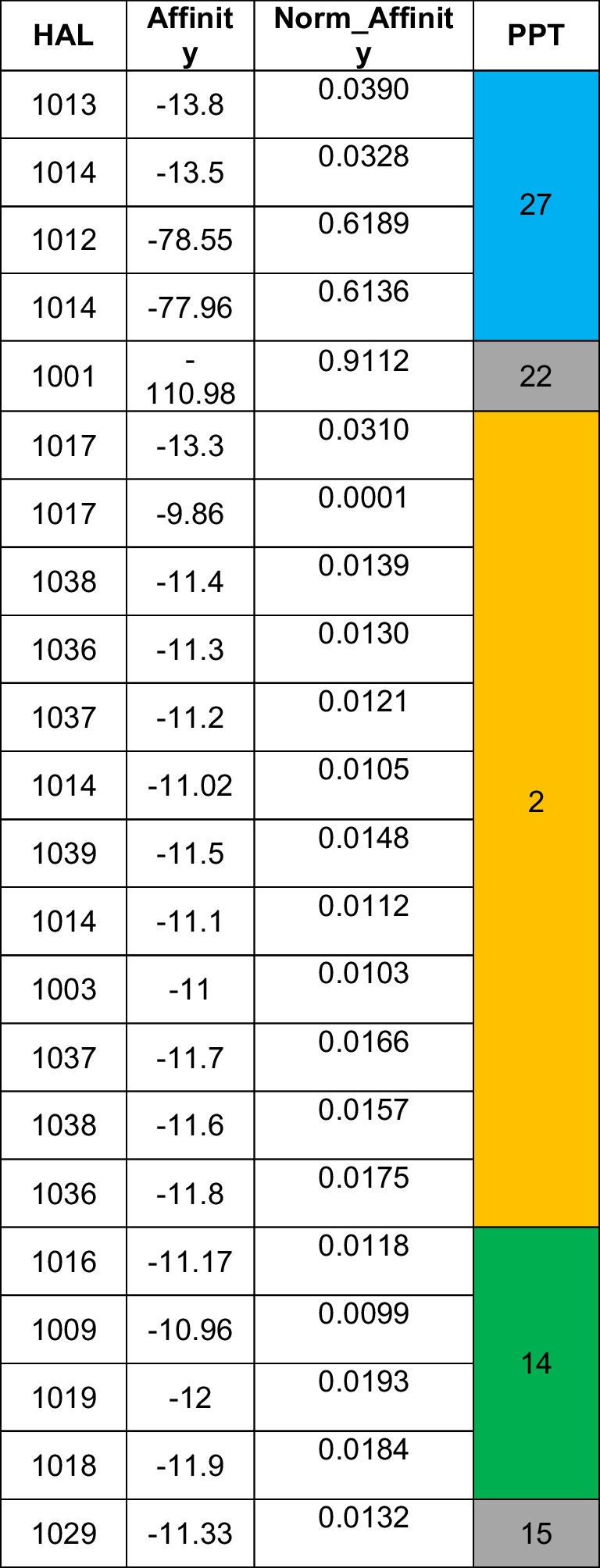
Final set of PPTs obtained based on HALs

It is evident that the PPTs are PPT2 (Rank 1), PPT 14 and PPT27 (Rank 2), and PPT15 and PPT22 (Rank 3) as three independent clusters. Ideally, the clustering should affect three big clusters — 2, 14, and 27, as is attempted in ML application below. It is important to note that ranking of PPTs need to be validated also. Hence, KMC, which is one of the most popular clustering techniques, is chosen as an efficient ML technique.

#### Dependency

**Fig. 1:**
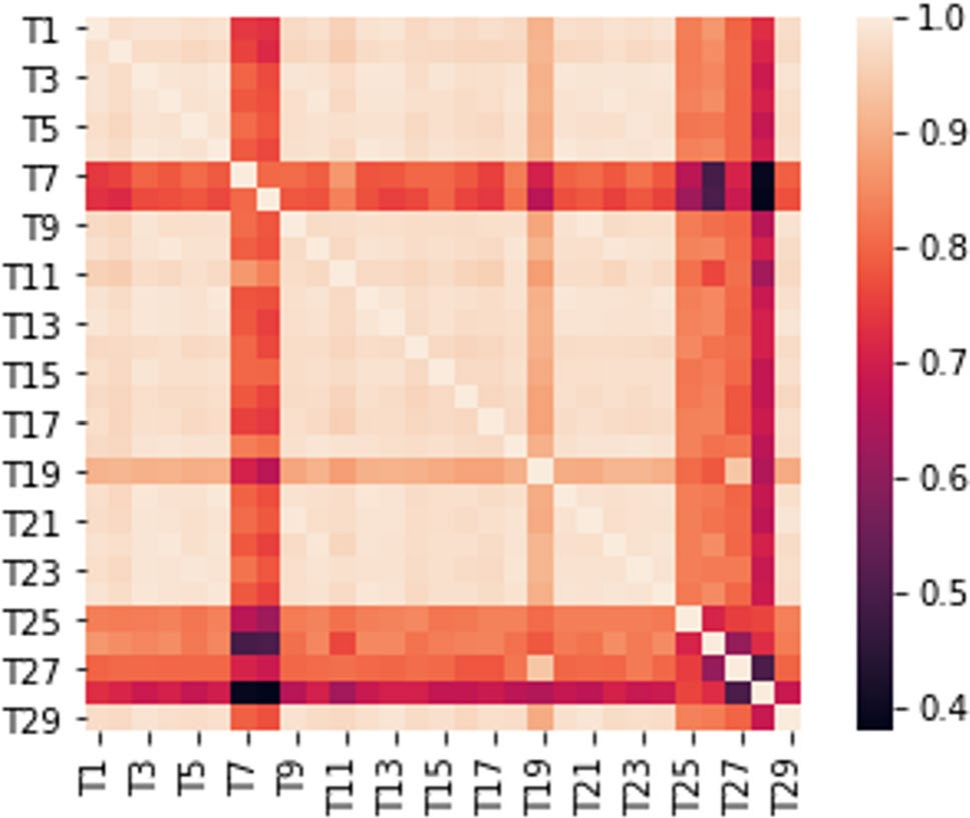
Correlation heatmap

It seems that target proteins are “dependent” on each other (Pearson’s correlation test = 0.970, *p*-value < 0.05), i.e., mostly linearly correlated (refer to Fig. 1).

##### Correlation heatmap

In Fig. 1, most PTs (indicated by Ts in the figure) show positive correlations with values close to 1 (0.970) as seen in the color trackers (heat map equivalent).

**Fig. 2:**
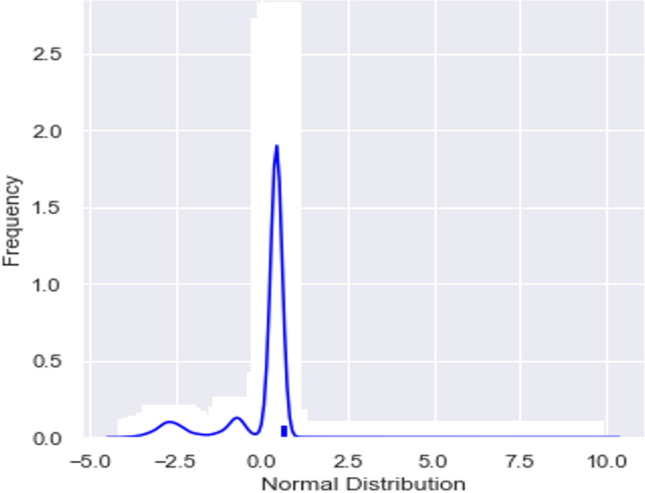
Distribution plot

Figure 2 is representative of PTs distributed over a normal distribution profile.

Distribution: None of the target proteins have symmetrical Gaussian distributions (Shapiro–Wilk test stat (*W*) = 0.560, *p* value < 0.05, CI = 95%).

Next, three clustering techniques, e.g., KMC, GMM, and DBSCAN, are used as the efficient and popular unsupervised machine learning (ML) techniques to cross-validate the results, especially the number of clusters obtained and noise (outlier) handling through the above-mentioned rigorous data mining exercise on HPC.

### Approach II: machine learning (ML) — for ‘automation’ and ‘reverse modeling’

The objective here is to test the correctness of manual data mining (DDM) results, accommodate the PPT outliers (PPT15 and PPT22 in Table 12), and then automate the process of predicting possible PPTs for a given set of test HALs. For this purpose, ML has been considered; more specifically, KMC has been chosen as one of the most popular clustering techniques [22–24]. From KMC, 3 good clusters (note, as indicated earlier, we already expected 3 clusters from max–min normalization) are targeted in line with the same number from DDM (refer to Table 12). Good clusters are defined as the ones with spherical conformation, that do not overlap, and have no outliers; i.e., all Ls can be accommodated within the clusters. Moreover, in this framework, KMC (an unsupervised ML method as the DUD-E data is unlabeled) can automate the PPT prediction process with reasonable accuracy. As mentioned above, two other clustering techniques, such as GMM and DBSCAN, are used to validate the output of KMC.

#### KMC: the steps are given below.

Step 1: Data scaling is done for 40 × 10, i.e., 40 APs each from 10 DB under study. Hence, 400 AP L-set is taken as the step for data wrangling/preprocessing.

Step 2: Calculating inertia to find the initial number of clusters. Essentially, it is the sum of squared error (SSE) for each cluster. Hence, the denser the cluster, the smaller is the inertia. Because inside the desired cluster data points are closest to each other, low values for inertia are meaningful.

**Fig. 3:**
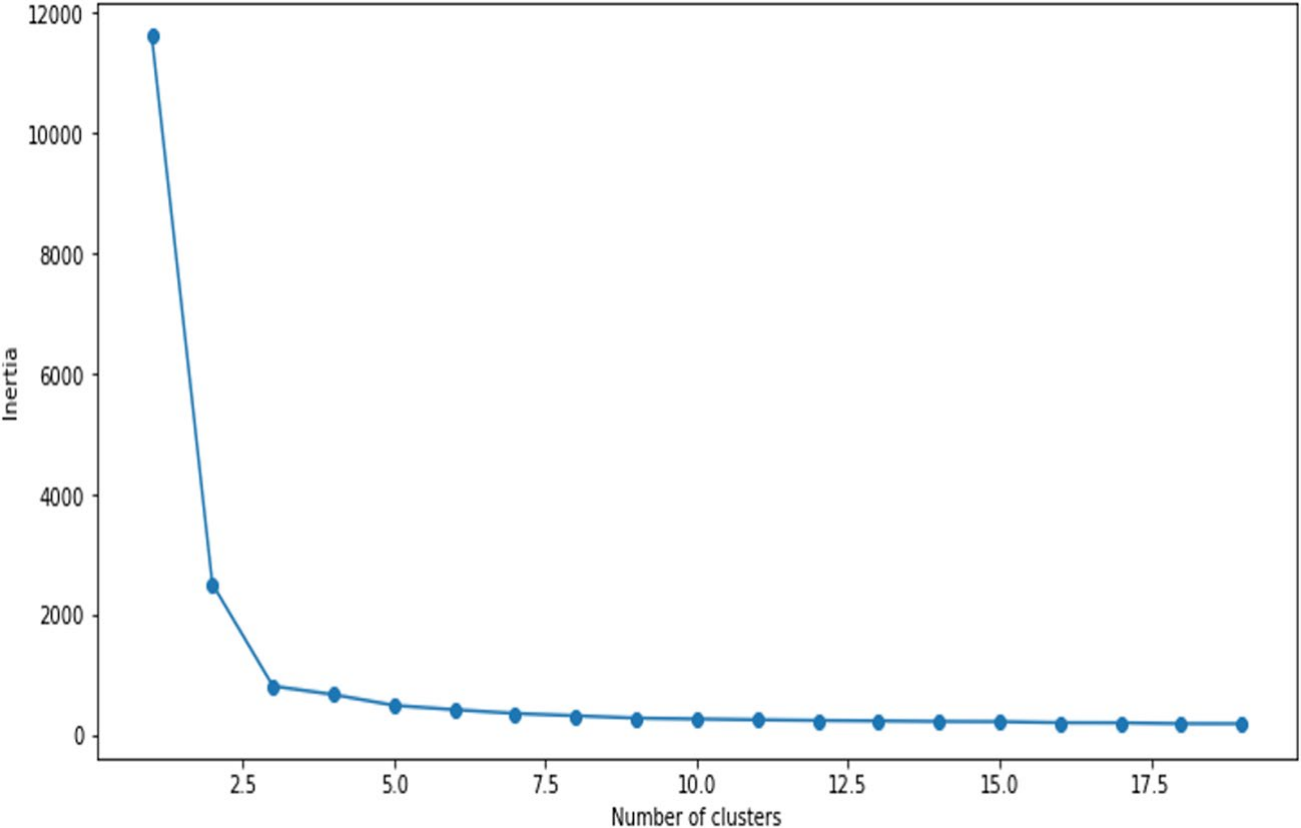
Inertia values to predict correct number of clusters

**Fig. 4:**
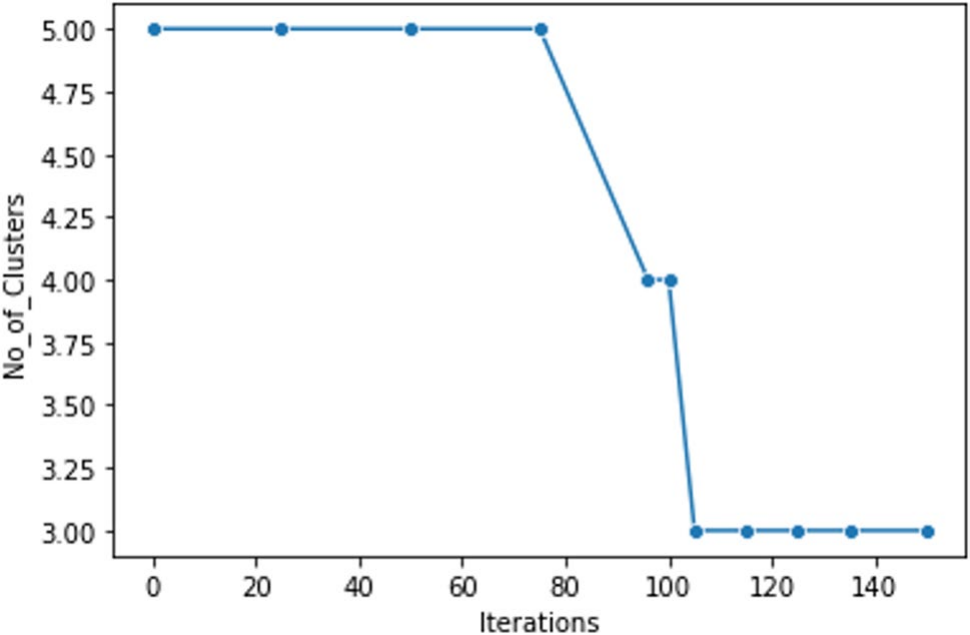
Iterations versus number of clusters. *Iteration 0th,* 5 clusters (0–4) and its corresponding number of Ls (total 400):

In Fig. 3, the calculated value of inertia, obtained iteratively over 3 clusters, shows up as 494.5 that is adequately low. However, across all 3 clusters, the step counts are monotonous with not much difference in inertia values. Therefore, the initial number of clusters is considered to be 5 and the aim is to further iterate towards a suitable convergence when all the data points are accommodated inside the final (reduced) number of clusters. The iterative convergence shown below assumes 5 initial clusters. Table 4 below shows the convergence rate of the clusters against the number of iterations performed (Fig. 4).

**Table:** 

1 320
2 40
0 31
3 **8**
4 **1**

Observation: One plus eight, i.e., total 9 data points, are considered as the outliers as because in comparison to other clusters, its counts are very low. Hence, the aim is to accommodate these outliers within their neighboring clusters.

Iteration 96th, 4 clusters:

**Table:** 

1 320
2 40
0 39
3 **1**

Observation: 8 earlier outliers are accommodated inside the first cluster. The fourth cluster still has one data point and is considered as an outlier. Our aim is to accommodate this into the neighboring cluster to get compact clusters without any outlier, an accepted quality assurance of any good clustering technique.

Iteration 105th, 3 clusters:

**Table:** 

1 321 (80.25%)
2 40 (10.00%)
0 39 (09.75%)

Observations: Remaining data points at the fourth cluster have been successfully accommodated into cluster 2. After this iteration, no further change in the number of clusters and corresponding Ls is found.

Summary: A set of 400 × 29 data matrix of “ligands affinity (LA)” (rows) and “active protein target (APT)” (columns) can be partitioned efficiently into 3 distinct (un-overlapped) spherical clusters without any outlier. Therefore, the quality of clustering is good.

Step 3: Centroid calculation (training data): These are final centroids as with further iterations, its values are not changing anymore (refer to Table 13):

**Table 13:** Centroids based on affinity magnitudes of 400 HALs corresponding to 29 PTs

	PT1	PT2	PT3	PT4	PT5	…	PT25	PT26	PT27	PT28	PT29
C1	0.4195	0.4167	0.4399	0.4326	0.4417	…	0.3712	0.2873	0.4120	0.2284	0.4381
C2	− 2.8644	− 2.8335	− 2.7851	− 2.8103	− 2.7481	…	− 2.3875	− 2.7718	− 2.1523	− 2.3501	− 2.7525
C3	− 0.5149	− 0.5237	− 0.7641	− 0.6784	− 0.8172	…	− 0.6065	0.4779	− 1.1833	0.5301	− 0.7832

Table 3 shows distinct values such as (− 2.***), (− 0.***), and (0.4***) for three clusters (Tables 14 and 15).

**Table 14:**
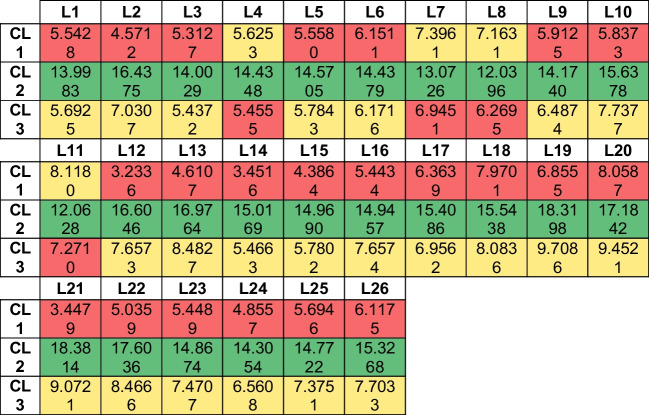
Euclidean distances of each of the 26 test Ls of test data ‘A’ from centroids of each cluster

**Table 15:**
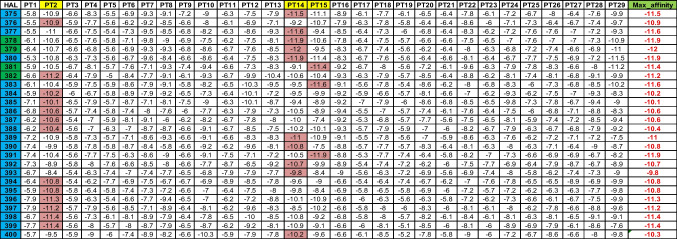
Mapping HALs to the corresponding PPTs — ‘reverse modeling’

**Fig. 5:**
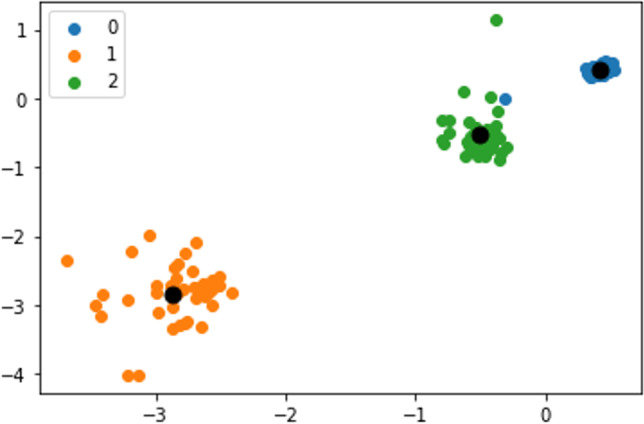
Three distinct clusters with centroids obtained with KMC

Step 4 (Visualization): 3 distinct clusters with centroids as black dots in Fig. 5. Here, “0” denotes cluster 1, “1” refers to cluster 2, and “2” signifies cluster 3.

It is interesting to see from the centroids thus obtained that cluster 2 contains most of the ligands (80.25%), followed by cluster 3 (10%) and cluster 1 (9.75%). This observation can be logically mapped to the findings from data mining that predict “high” affinity ligands with tendency to bind with APT2 (63%), APT14 (19%), and APT27 (18%). Hence, it can be safely concluded that cluster 2 is probabilistically expected to contain the maximum number of high affinity ligands (HALs) towards APT2. Cluster 3 is the next candidate with the maximum number of high affinity ligands pointing towards APT14, while cluster 1 data points are high affinity ligands targeted for APT27. We can conclude that of the 29 APTs considered, these three are the prime protein targets (PPTs). This observation needs to be validated by going back to the original test data set, which has been performed below (refer to Tables 16, 17 and 18). It should be borne in mind that for other L-sets, these PPTs may vary.

**Table 16:**
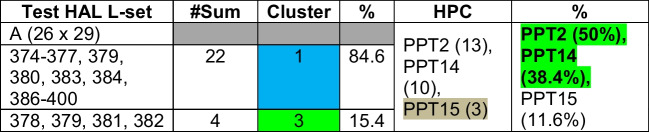
Validation of relationships among HAL test data “A” and PPTs based on clusters

**Table 17:**
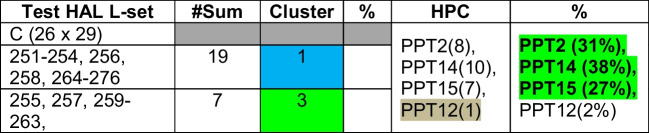
Validation of relationships among HAL test data “B” and PPTs based on clusters

**Table 18:**
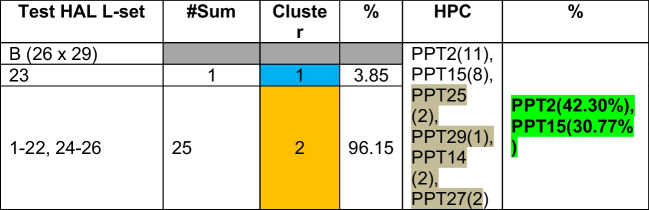
Validation of relationships among HAL test data “C” and PPTs based on clusters

Step 5: Validation on test L-sets (A, B, C), i.e., the reverse modeling:

Test L-sets have been selected from the given 400 HAL DB randomly — first 26 Ls from the “tail” of HAL test data (L-set A), second set of 26 Ls from the “middle” portion of the HAL test data (L-set B), and third set of 26 Ls are taken from the “head” of the HAL test DB (L-set C).

Euclidean distances (ED) of each of the ligands from the centroids have been computed. Each ligand is clustered (refer to Table 14) based on minimum distance (red colored cells) from the centroid. Using maximum affinity as the descriptor, HALs are mapped against the original data to identify the corresponding PPTs (refer to Table 15) and then evaluated for possible PPTs within the cluster, as shown in Tables 14 through 16. Table 15 shows corroborating HPC obtained by clustering (refer to Table 16).

Other HPC obtained by test data “B” and “C have also been corroborated in the similar way.

From the above experiments, we conclude that PPT2 (average HPC is 41.1%) is the highest ranked protein target as most HALs show high affinity towards it. PPT2 is followed by PPT14 (average 25.46%), and then PPT15 (average 23.12%).

Prime HAL information: (test set “A” — 26 data (375–400) picked from the tail of 400 total ligands; test set “B” — 26 data (251–276) picked from the middle portion of 400 total ligands; and test set “A” where 26 data (1–26) picked from the head of 400 total ligands.

Test set “A”: Ligand numbers 379, 380, 381, and 392 (15%) have maximum affinity towards PPT 14 (11%) and 15 (4%), respectively.

Test set “B”: Ligand numbers 259, 260, and 261 (11%) have maximum affinity towards PPT 14.

Test set “C”: Ligand numbers 12, 14, and 17 (11%) have maximum affinity towards PPTs 27, 27, and 2, respectively.

Therefore, out of 26 × 3 = 72 test ligands, 14% are found to be HALs, which gives a clue to the percentage of HALs that can be obtained from any number of ligands, which is another outcome of this work. For obvious reasons, though, the percentage may vary with the dataset.

It is important to note that the above analysis cannot qualify for the ranking of HALs as well as PPTs. It can only predict the key HPCs numerically and cannot argue for qualitative ranking, which requires domain expertise and in-vitro/vivo experimental analysis of individual HPCs.

Below, we validate the performance of KMC using two other clustering methods, GMM and DBSCAN.

#### The GMM clustering method

Working principle: It assumes that all data points originate from a finite number of Gaussian distributions with unlabeled/unknown parameters. Hence, it is a probabilistic unsupervised ML model.

Observation: The clusters are plotted similarly as KMC to retain visual uniformity (see below figure). The blue colored dataset makes cluster 1 (contains 321 datasets), while clusters 2 and 3 are represented in yellow and green colors, respectively, containing 40 and 39 datasets. The centroid properties are also identical to KMC. It is important to note that similar to the KMC method, cluster 2 is probabilistically expected to contain the maximum number of HALs towards APT2. Cluster 3 is the next candidate with the maximum number of HALs pointing towards APT14, while cluster 1 data points are high affinity ligands targeted for APT27. We can conclude that of the 29 APTs considered, these three are the prime protein targets (PPTs) as already identified by KMC.

**Fig. 6:**
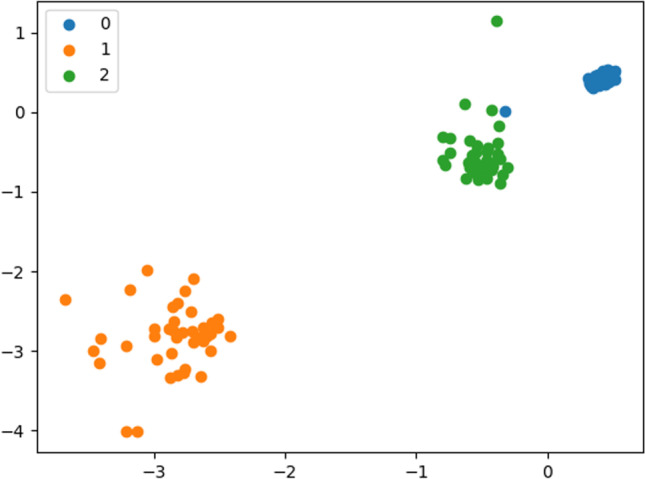
Three distinct clusters with centroids obtained by GMM method

Summary: A set of 400 × 29 data matrix of LA (rows) and APT (columns) can be partitioned efficiently into 3 distinct (un-overlapped) spherical clusters without any outlier using GMM method (Fig. 6).

#### The DBSCAN clustering method

Working principle: The algorithm identifies the group of data points based on the assumption that the data points inside the respective cluster belong to distinct contiguous density of higher priority points that are separated from a distinct contiguous density of relatively lower priority points. The algorithm can cluster a high-volume dataset having errors and noise within the dataset and thus algorithmically superior to KMC and GMM algorithms. Moreover, DBSCAN does not require initialization of cluster number and thus do not come across over and under-fitting issues of clustering and faster than that of KMC and GMM. Since KMC and GMM may cluster low-priority data points, alongside GMM, we have implemented DBSCAN for cross-validation of KMC and GMM-based outputs.

**Fig. 7:**
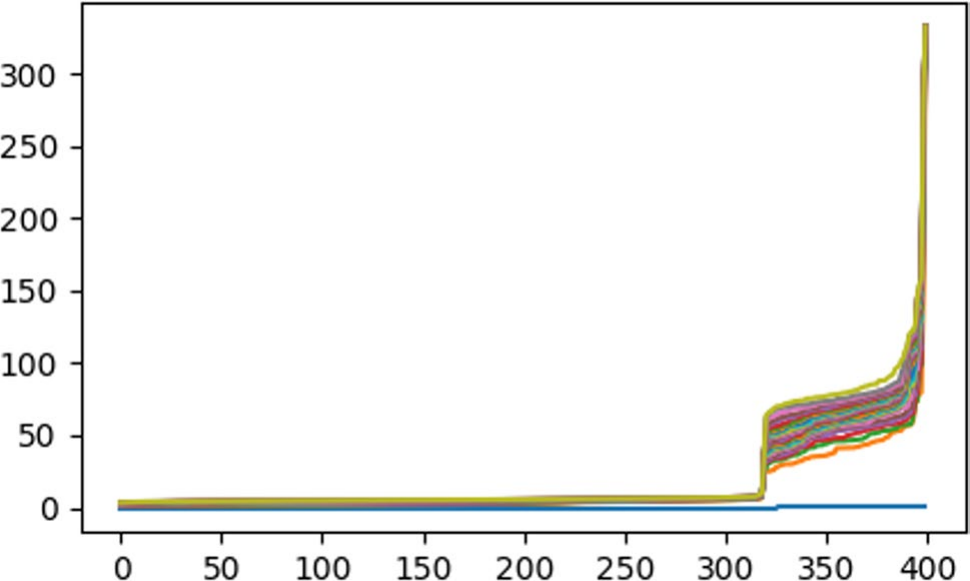
The ‘eps’ value obtained by DBSCAN method

DBSCAN requires two parameters: (i) “epsilon (eps),” the least distance between two neighboring points, and (ii) “MinPoints (Mpt),” the minimum number of data points required to construct a cluster. For our data points, “‘Mpt” is calculated as 2* data dimension, i.e., 2 * 29 = 58, while “eps” is calculated from the distance plots (refer to Fig. 7). From the figure, it is noted that maximum curvature (least distance) occurs at 96 (refer to the y-axis), which is our “eps” to run the algorithm.

**Fig. 8:**
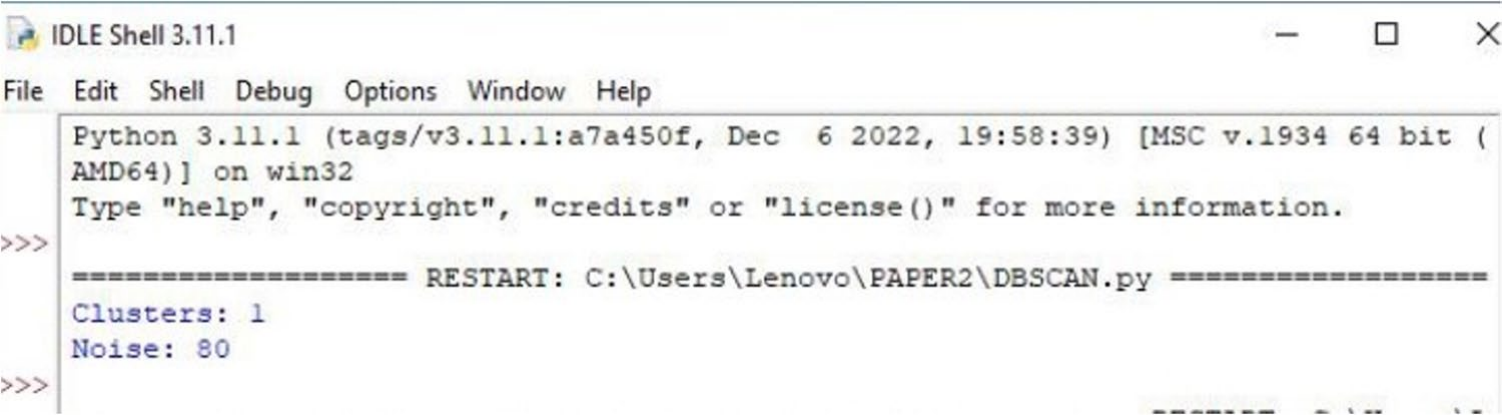
Number of clusters and noise for the dataset printed on Python 3.11.1 IDLE Shell installed on 06/12/2022 in the win32 64 bits system

Observation: With the above “eps” and “‘Mpt” values, DBSCAN can yield one cluster but there are 80 outliers. It is most probably due to varying density among the data points within our high-dimensional data. Hence, we have discarded DBSCAN in this work (see Fig. 8).

## Discussions

Ten years is a typical gestation time for a new drug to hit the market. Clinical trials alone take six to seven years on average. The average cost towards each successful drug is estimated at $2.6 billion [23] (http://phrma-docs.phrma.org/sites/default/files/pdf/rd_brochure_022307.pdf). The failure rate of a new drug to reach the market is around 88%, which means only 12% of projected drug candidates are eventually marketed as genuine drugs (http://phrma-docs.phrma.org/sites/default/files/pdf/rd_brochure_022307.pdf), notwithstanding such high expense. Failure can happen due to various causes starting from a wrong choice of PTs and Ls and its combinations at the experimental stage in the laboratory to regulatory stringencies and finally adoption by the healthcare workers and the end users. Any successful new drug must have high efficacy, low dosing, rapid actions, and only a few side effects. It should also be able to reduce the morbidity load, cost of hospitalization, and curb mortality. The key to time and cost conducive delivery is thus fast and accurate identification and validation of PPTs and the HALs that can efficiently combine with each other to give a stable molecule that helps designing an effective drug. This is where intelligent, machine learned, molecular docking can make the crucial difference between success and failure, and certainly in taming cost.

This work is an attempt to detect PPTs for a given sample of HALs on 10 L-sets, each obtained from standard DOCKING programs. The approach complements a recent benchmark [21] where a novel statistical combination, popularly called consensus scoring (CS), was used to predict the PPTs for the same dataset. The present independent approach provides a validation of the outcomes from that work as also in terms of being risk validated itself from this verification. DDM is done on ALs (decoys are discarded), and based on their individual receptiveness to Ls or agents, our probabilistic model predicts *PPT2 (55%)*, *PPT14 (19%)*, *PPT27* (18%), *PPT15* (4%), and *PPT22* (4%) as the most promising PTs out of 29 choices; i.e., only 17% of the PTs show high receptiveness as prime targets to the agents.

As the DB is unlabeled, KMC has been applied as an ML technique to test the efficiency of the above DDM approach. KMC can produce 3 distinct clusters. To validate the observations of DDM, the neighborhood of each ligand in all three test samples is measured from the centroids of each cluster. It is evident that *PPT2* (average possibility of getting stable HPC is 41.1%) is the highest ranked among all, as most HALs show high affinity towards it; followed by *PPT14* (rank-2 average 25.46%), and then *PPT15* (rank-3 average 23.12%), respectively. The result is further validated by GMM and found to be similar as KMC. Thus, KMC provides a rigorous DDM approach that can be automated to generate faster and accurate drug prediction routines. Importantly, for pursuing this approach, large training samples are not necessary as this is a “sparse classifier.” This method can be used in new sets of Ls-PTs from DB to identify PPTs towards successful laboratory tasting of real drugs.

### Advantages of the method:

- The algorithm does not require large training (macro-supervised learning not needed) of DB due to its efficient redundancy handling algorithm around the maximum of the mean affinity.
- DDM and KMC-based ML complement each other in terms of accuracy and speed, thus, reducing the time taken to discover right PTs/drug candidates.
- The method does not require complex computations.
- This method can efficiently handle sparse unlabeled data having noise.
- This method is also cost-efficient as it only requires moderate computation, not chemical samples.

### Limitations of the work and hence the targeted future research are as follows:

- This work focuses only on ALs; decoys are discarded. In future, similar approaches can be adopted even for the decoys to validate whether these agents are genuine decoys.
- Other clustering techniques (such as fuzzy C-means clustering (FCM) technique) could also be used in identifying PPTs, which have overlapping binding features with Ls.

## Data Availability

Open-sourced protein and ligand data have been used from DUD-E repositories (http://dude.docking.org). Codes, written respectively in Matlab_R2021a and python3.8, could be made available on request.
